# Supplementary material for: MetaRibo-Seq measures translation in microbiomes
Source: Nat Commun. 2020 Jun 29;11:3268. doi: 10.1038/s41467-020-17081-z (PMC7324362; doi:10.1038/s41467-020-17081-z)
Supplement: Supplementary file 10 — Supplementary Data 7 [file 41467_2020_17081_MOESM10_ESM.zip › File2/Confidence_VeryHigh_Taxonomy/228785_out.krona.html]

Javascript must be enabled to view this page.

members
magnitude
magnitudeUnassigned
count
unassigned
taxon
rank

228785\_out

6

superkingdom
2
6

phylum
1239
4

1
909932
class

1843489
order
1

1
family
31977

1
29465
genus

1

SRS047126\_contig\_number\_1248
species
450749

186801
class
2


SRS023715\_contig\_number\_contig-100\_34649.34650
186802
1
2
order

186803
family

SRS062427\_contig\_number\_contig-100\_9969.9969
1

class
91061
1

order
186826
1

186827
family
1

46123
genus
1

46125
species

SRS042691\_contig\_number\_7377
1

phylum
976
1

class
117743
1

1
200644
order

1
49546
family

genus
1016
1

SRS020226\_contig\_number\_contig-100\_4091.77435

1
544448
phylum

31969
class
1

1
order
186329


SRS053356\_contig\_number\_contig-100\_12834.181488
1
2053494
species
